# Diagnosis of Prostate Cancer: A Comparative Evaluation of Biological Techniques

**DOI:** 10.3390/diseases14060217

**Published:** 2026-06-17

**Authors:** Unathi A. Tshoni, Thokozani P. Mbonane, Phoka C. Rathebe

**Affiliations:** Department of Environmental Health, Faculty of Health Sciences, Doornfontein Campus, University of Johannesburg, P.O. Box 524, Johannesburg 2006, South Africa; tshoniunathi@gmail.com (U.A.T.); tmbonane@uj.ac.za (T.P.M.)

**Keywords:** prostate cancer, diagnosis, screening, biomarkers, specificity, sensitivity

## Abstract

Prostate cancer (PCa) is the most prevalent cause of cancer-related deaths worldwide. This review aims to synthesize the contemporary literature (2018–2026) on various diagnostic approaches of PCa for clinicians. Currently, the standard approach to PCa diagnosis includes (1) prostate-specific antigen (PSA) testing, (2) its derivatives, and (3) digital rectal examination (DRE). This approach is readily available and cost-effective but lacks specificity, thus resulting in a high incidence of overdiagnosis and overtreatment of PCa patients. This review aims to compare the traditional approaches with the newly developed approaches to PCa diagnosis and investigate their suitability for research purposes, including urine-based markers, liquid biopsy, and histopathology. Each of these approaches has advantages and disadvantages. Advanced imaging techniques like multiparametric magnetic resonance imaging (mpMRI) are helpful in the localization of PCa and can prevent unnecessary biopsies. Histopathology remains the gold standard in the confirmation of PCa diagnosis. Newly developed blood-based markers and advanced imaging techniques are more sensitive and specific in the diagnosis of PCa and are likely to result in a better clinical outcome than the traditional approaches. However, the cost of these approaches remains a global challenge in developing and developed countries alike. PCa diagnosis tools and strategies are presented in a clinical and cost-effective manner and are integrated in a tiered approach with the aim of controlling and managing PCa and its diagnosis.

## Simple Summary

This paper offers a comparative analysis of the various prostate cancer diagnostic tools based on cost-effectiveness, invasiveness, accuracy, and suitability for research purposes. Low-cost and readily available diagnostic tools like PSA tests and DRE tests are effective for mass screening procedures due to low costs, but their lack of specificity and high chances of overdiagnosis make them less desirable. High-tech tests such as biomarker-based, multiparametric MRI, and liquid biopsy tests are better suited for diagnosis as they have higher accuracy and more informative data, but their expensive and non-wide availability limit them from general use, especially in underdeveloped countries.

## 1. Introduction

Prostate cancer is a very serious health issue worldwide [1,2]. It has the highest rate of diagnosis as cancer among males and comes second in ranking for causing cancer deaths in this population [2,3]. It is still one of the top three cancers diagnosed among men in Africa [4,5,6]. This fact shows how much the disease can affect male health and wellness, making it important to be aware of it and catch it early to lessen its connected impact [1,7,8]. The disease remains a significant threat to men’s health globally but poses particularly pressing issues within an African context [5,9]. The genetic basis of the high prostate cancer risk among men of African ancestry is only beginning to be understood [10,11]. Multiple inherited and somatic genetic variants associated with increased risk of developing the disease or of aggressive disease have been identified in large studies. Inherited risk variants include those influencing androgen signaling and tumor biology, while somatic gain-of-function mutations in androgen receptor and PTEN cooperate to drive malignant progression of prostate tumors [5,12,13,14].

This review gives detailed information about clinical services for prostate cancer in South Africa and globally, and it also places the country both as a national and international leader in this area while challenges exist regarding improving cancer outcomes [6,9,15]. Overall regional epidemiological trends reveal that prostate cancer incidence rates are significantly lower throughout Africa than those reported in South Africa [6,16,17,18]. These findings are further corroborated by an analysis of population-based data showing a marked variation in incidence across the African continent. The data from 30 of 54 recognized countries note a median reported age at diagnosis as being 58 years and substantiate further understanding evolution and surveillance needs for prostate cancer within an African context [4,5,6].

Ongoing emphasis, education, and treatment options are very important in facing prostate cancer as a disease [1,14,19,20]. Biological diagnostic techniques like gene tests and biomarker measuring are the main ways of gaining knowledge [21,22,23]. Diagnostic steps are very important in balancing care and therapy hence critical in reducing early death [14,24]. Early detection and care boost survival chances from below 30 percent to nearly 90 percent [25,26]. Traditional diagnosis relies on serum prostate-specific antigen levels, digital rectal examination, and ultrasound guidance for biopsy. However, PSA has low specificity which leads to unnecessary biopsies [27,28,29]. Diagnosis is generally confirmed through histopathological examination of the biopsy sample, considered the gold standard, though it can be associated with some complications due to its invasive nature [30,31]. Hence, there has been a demand for effective, non-invasive, accurate diagnostic tools [32,33,34].

This study will review the literature on the various biological methods used in the diagnosis of prostate cancer, with emphasis not only on the availability of resources but also the complex structures of health systems that support these diagnostic activities. It is part of a larger effort to respond to relevant research questions that are interconnected with prostate cancer and its effects on patient management, and more specifically, an attempt at gathering and synthesizing information that can assist the South African Diagnostic Research Initiative in improving or upgrading its activities and techniques in this domain. By putting such insights together, we are aiming at making very significant contributions towards improvements in approaches to diagnostics within the context of research and treatment dealing with prostate cancer, thereby paving pathways for better outcomes for patients. The goal here is to facilitate a deeper understanding of the empirical data and build stronger frameworks securing efficient non-invasive diagnosis and treatment processes.

## 2. Methodology

This study used a narrative literature review design to discover and synthesize information on prostate cancer diagnostic techniques, particularly the less invasive ones.

### 2.1. Information Sources and Search Strategies

A comprehensive literature search was carried out on electronic databases including PubMed, Scopus, Web of Science, and Google Scholar. The objective was to obtain data regarding both established and emerging diagnostic methods from recent literature (2018–present). A combined approach of Medical Subject Headings (MeS–H) terms with free-text keywords was utilized. The principal search terms were: “prostate cancer diagnosis,” “prostate cancer screening,” “prostate-specific antigen (PSA),” “urine biomarkers prostate cancer,” “liquid biopsy prostate cancer,” “multiparametric MRI prostate cancer,” “prostate biopsy techniques,” “prostate cancer Africa,” and “prostate cancer South Africa.”

### 2.2. Eligibility Criteria

Studies published between 2018 and 2026 were considered for inclusion if they were peer-reviewed articles focusing on research about diagnostic techniques for prostate cancer, providing data on sensitivity or specificity or clinical application or cost metrics involving African populations or global studies concerning Africa.

Exclusion criteria comprised studies not focused directly on diagnostics, studies focused exclusively on treatment, non-English publications, editorial case reports, editorials with insufficient methodological rigor, and studies that do not provide adequate data on diagnostic performance.

### 2.3. Article Selection Process

**Figure 1:**
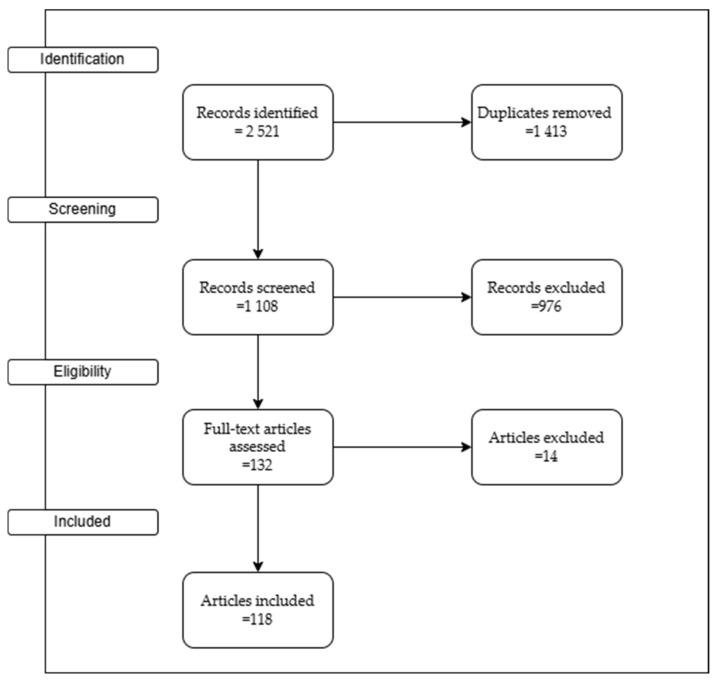
PRISMA flow diagram for studies included in this review.

Titles and abstracts were screened for relevance. Full-text assessments were then undertaken to determine eligibility based on the inclusion criteria. Priority was given to (1) systematic reviews and meta-analyses, (2) large cohort and case–control studies, and publications on diagnostic accuracy (sensitivity and specificity). The Preferred Reporting Items for Systematic Reviews and Meta-Analysis (PRISMA) framework was used. The flow diagram in Figure 1 summarizes the identification, screening, eligibility of full-text articles assessed, inclusion, and exclusions at each stage of the study selection process. The reasons for exclusions were: at the identification stage, articles were excluded because they were duplicates; at the screening stage, articles were excluded because they were non-English publications, not relevant to the field of diagnostic methods and published before 2018; and at the eligibility stage, articles were excluded because of insufficient data among the reported outcomes. This review has been submitted to the International Prospective Register of Systematic Reviews (PROSPERO) platform for registration (CRD420261400728).

There was no comprehensive search for gray literature sources; only articles from peer-reviewed databases were used to reduce publication bias. Subsequently, all the included studies were published in the English language and only had human participants.

Multiple appraisal tools were used to evaluate methodological rigor and assess quality and risk of bias because of heterogeneity in study designs of the included papers. The tools used are listed in Table 1 along with their applicability; highlighting studies assess, methodological criteria and types of study designs. Each tool was selected based on the methodological traits and objectives of study. (1) The Joanna Briggs Institute Critical Appraisal Tool (JBI) was chosen to evaluate the methodological quality and risk of bias of the included review (systematic, the literature, scoping and commentary) studies. Depending on the particular study design under review, different JBI appraisal checklists were used. Participant selection, measurement reliability, confounding variables, thoroughness of outcome reporting, and suitability of statistical analysis were among the methodological areas that were the focus of the evaluation. (2) The Quality Assessment of Diagnostic Accuracy Studies (QUADAS-2) tool was used to evaluate studies that assess the diagnostic efficacy of prostate cancer screening techniques. This tool evaluated quality across multiple variables such as index test perfomance, reference standards, patient selection, flow and timing to determine if sensitivity and specificity were accurately estimated. A substantial portion of this research is focusing on comparing diagnostic performance of various techniques, hence this tool is the best for assessment. (3) The Newcastle-Ottawa Scale was used for observational (cohort, comparative clinical, epidemiological and case–control) studies because these explored incidence of prostate cancer, racial disparities, biomarker linkages and clinicopathological features among various populations. This tool assesses participant selection, study group comparability, and the sufficiency of outcome or exposure assessment. So it was chosen to assess the quality of non-randomized studies, making it possible for the review to assess the validity of observational data on the prevalence, course, and population-specific risk factors of prostate cancer.

According to the QUADAS-2 assessment, most of the diagnostic accuracy studies showed moderate to high methodoligical quality with a typically low risk of bias across domains. Histopathology was frequently employed as the diagnostic reference standard in studies assessing PSA derivatives, PHI, PCA3, TMPRSS2-ERG, SelectMDx, ExoDx, mpMRI, PSMA PET/CT, and MRI-targeted biopsies, enhancing diagnostic reliability. For instance, when assessing PSA, DRE, PSMA PET/CT, and mpMRI performance, Merriel et al. (2022), Matsukawa et al., Satapathy et al., and Yang et al. [29,46,74,80] showed excellent methodological rigor and consistent diagnostic techniques. In a comparable manner, urinary biomarkers like SelectMDx and ExoDx were robustly evaluated by Hendriks et al., Margolis et al., and Wu et al. [56,57,60] to reduce needless biopsies. However, due to smaller sample sizes, little external validation, and variable diagnostic thresholds, some new biomarker studies, such as Koo et al. and Warli et al. [43,44] showed a moderate or unclear risk of bias. All things considered, the QUADAS-2 results confirm the increasing diagnostic value of sophisticated molecular and imaging techniques over conventional PSA-only screening techniques.

Based on the NOS assessment tool, these studies demonstrated a moderate to high methodological quality, with most of them getting a rating between 7 and 9 stars. Studies like those by Babajide et al. and Choi et al. [90,94] presented biologically relevant findings about biomarker and circulating tumor cell performance in prostate cancer populations. Parrallel to this, Martins et al. and van der Slot et al. [95,97] strengthened confidence in imaging and pathological findings by exhibiting strong methodological techniques in assessing mpMRI diagnostic localization and histological grading variability, respectively. Additionally, Tolkach et al. [96] showed significant external validation of artificial intelligence algorithms for Gleason grading across many institutions, enhancing the repeatability and dependability of automated pathological evaluation.

The JBI appraisal of review based studies indicates that most of these studies were helpful for qualitative synthesis given they clearly discussed prostate cancer diagnostic techniques including PSA, urine biomarkers, liquid biopsy, mpMRI, PSMA PET/CT, biopsy techniques and digital pathology. Notable reviews offered targeted remarks on diagnostic methods and supported their findings with data; showing that routes that combine biomarkers, imaging and targeted bipsy are replacing singular diagnostic techniques like PSA [34,106,131,149,150]. However, the results also point to certain methodological shortcomings. Several reviews [129,133,134] were helpful for clinical context and background information but they lacked thourough reporting of study selection or formal critical appraisal procedure, which may result in selection bias and reporting prejudice because it is unclear if all pertinent material is methodically found. Similarly, reviews that highlight cutting-edge technologies like liquid biopsy and exosomes offered valuable insights but frequently highlighted promising future applications while acknowledging limited standardization and clinical validation. Overall, the JBI-evaluated literature proved useful for mapping current understanding and spotting trends, particularly in the areas of imaging, precision diagnostics, and non-invasive biomarkers.

Several recurrent methodological biases and limitations were found in diagnostic accuracy studies, observational studies, and review-based evidence. Selection bias was one of the most common biases found, especially in observational and diagnostic research evaluated using NOS and QUADAS-2. Patients from high-risk clinical populations or specialist tertiary care facilities were included in a number of biomarker and imaging studies, which may have overstated diagnostic performance and decreased generalizability to larger screening populations. The likelihood of sampling bias was increased by the use of relatively small or highly selected cohorts in studies like that of Warli et al. [44].

To reduce subjectivity and reviewer-related prejudice, two reviewers separately assessed the risk of bias. After each reviewer assessed the included studies independently, the findings were compared, and disagreements were settled by consensus and discussion. The screening and risk of bias assessment processes did not make use of any automation technologies, artificial intelligence softwre, or machine learning programs. Based on the evaluation results, the overall quality of the studies was classified as low, moderate, or high risk of bias.

**Figure 2:**
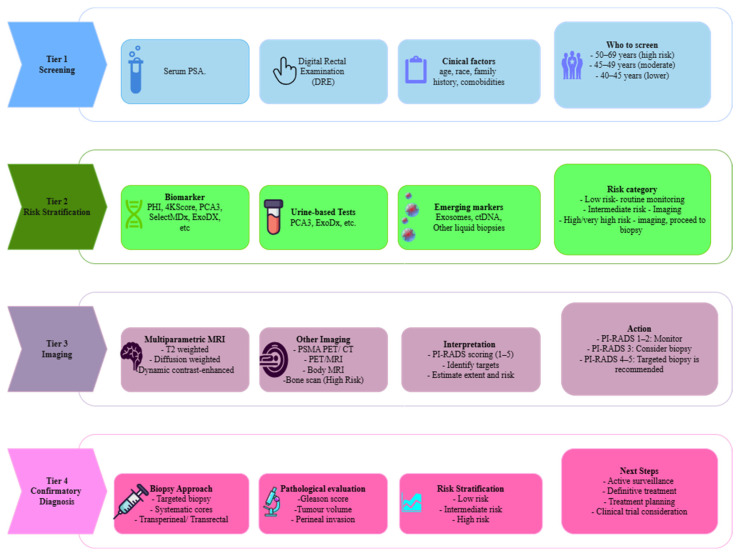
A tired diagnostic pathway for prostate cancer.

Figure 2 presents a tiered diagnostic pathway illustrating sequential use of diagnostic tools from screening to diagnosis. The pathway is organised into progressive tiers; each representing an increase in diagnostic specificity, resource requirement and complexity. The first tier shows initial screening and risk assessment; seconf tier showing secondary risk stratification tests; third tier represents imaging techniques for localising and charectarising disease and the fourth tier showing disease confirmation.

### 2.4. Data Extraction

Relevant data were carefully extracted in tabular form. The extracted variables included: method of diagnosis, sample type (blood, urine imaging tissue), sensitivity, specificity clinical use (screening or confirmation), and approximate cost level of invasiveness.

### 2.5. Data Analysis

A qualitative synthesis was used to compare the methods of diagnosis. These results are summarized in a table under three heads: (a) less invasive methods (e.g., PSA, urine biomarkers, liquid biopsy), (b) imaging methods (e.g., TRUS, multiparametric MRI), and (c) confirmatory methods (e.g., biopsy and histopathological analysis). Emphasis was placed on those most often used in Africa and South Africa taking into account cost accessibility and current healthcare infrastructure.

## 3. Results

The diagnostic techniques shown in Table 2 have been systematically developed from basic screening techniques to more advanced risk-stratified molecular approaches with their specific clinical applications and limitations for each step along the way. Conventional methods such as digital rectal examination (DRE) and prostate-specific antigen (PSA) testing—a protein produced by prostate cells and measured in blood as a biomarker for prostate cancer screening [35,98,99]—continue to be the mainstay of initial screening largely because they are widely available.

Several derivatives of PSA—free/total PSA ratio, PSA velocity, and PSA density—have been developed in an attempt to improve the discrimination between benign and malignant conditions particularly within the gray area of PSA values. More advanced blood-based assays like the Prostate Health Index (PHI)—a blood-based biomarker that combines multiple PSA derivatives to improve the detection of clinically significant PCa [45,46]—and 4Kscore test—a blood-based diagnostic test combining PSA derivatives with kallikrein protein biomarkers with clinical information to estimate the probability of aggressive PCa [50,51,52,53,54] to provide better risk stratification by predicting clinically significant aggressive cancer thereby reducing unnecessary biopsies.

Urine-based tests such as PCA3—PCa-specific non-coding RNA that is overexpressed in prostate cancer tissue and detected mainly in urine [37]—and TMPRSS2–ERG—a common genetic alteration in prostate cancer, associated with prostate tumor development [59,60]—improve specificity through molecular detection; however, the latter is limited by its lower sensitivity since it is present only in a small subset of prostate cancer cases. Next-generation urine assays like SelectMDx and ExoDx have a considerable clinical benefit in biopsy avoidance for a large portion of patients which indicates a larger shift toward non-invasive patient-centered diagnostics.

On the other hand, high-level diagnostic tools like liquid biopsy, imaging, and tissue-based methods emphasize improving precision, describing the illness, and confirming findings. Parts of liquid biopsy which are circulating tumor DNA, circulating tumor cells, and exosomes give important molecular details and allow non-invasive checking though their sensitivity may change based on disease stage and sample origin. Imaging methods help to add to these strategies with transrectal ultrasound and are mainly used as a guide for biopsy while multiparametric MRI improves detection through standardized scoring systems like PI-RADS. PSMA (Prostate-Specific Membrane Antigen) PET/CT imaging is a newer advanced non-invasive method that can offer detailed anatomical and functional visualization especially in more advanced cases of this disease.

Table 3 summarizes the considerable variability in the diagnostic accuracy of prostate cancer diagnostic modalities with respect to their clinical application and type of sample. The pooled sensitivity and specificity estimates are reported for all studies that were included in the literature review. Moderate to low sensitivity has been reported for screening modalities, namely digital rectal examination (DRE) and prostate-specific antigen (PSA) testing (DRE: 28.6–53.2%; PSA: 21–44%), with relatively higher specificity values, especially for PSA (91–94%). These results indicate that while such methods may be useful for preliminary diagnosis, many true positive cases could be missed by these tests, particularly when the disease is at an early stage.

PSA derivatives, including free/total PSA ratio and PSA velocity/density, have better diagnostics parameters with moderate sensitivity and specificity; hence they are recommended for risk stratification and follow-up rather than being used as stand-alone diagnostic tools. More advanced blood-based assays like the Prostate Health Index (PHI) test or 4Kscore can achieve very high sensitivities (up to about 95–97%) but have relatively low specificities; this means that while such tests may be good at finding potential cases of prostate cancer, they increase the risk of overdiagnosis when used alone.

Urine-based biomarkers and molecular assays have significantly improved the specificity of diagnosis, notably exemplified by the TMPRSS2–ERG gene fusion test, which achieves a specificity rate of up to 100% albeit with reduced sensitivity. Other assays like PCA3 and SelectMDx/ExoDx show moderate to high sensitivity and therefore serve as important tools for risk stratification and biopsy decision-making. Innovative liquid biopsy methodologies encompassing circulating tumor DNA (ctDNA), circulating tumor cells (CTCs), and exosomes demonstrate exceptional specificity often exceeding 85–100% in advanced disease cases; however, their sensitivity can be quite variable depending on the disease stage underscoring their primary use in monitoring and prognostic assessment rather than early detection.

Imaging modalities multiparametric MRI (mpMRI) plus advanced ultrasound techniques such as micro-ultrasound (MicroUS) plus artificial intelligence-enhanced transrectal ultrasound (TRUS) have reached a level of diagnostic accuracy whereby sensitivity rates could be approximately 90% or more; however, specificity may vary. PSMA PET/CT scans perform exceptionally well in detecting metastases as well as recurrences with both sensitivity and specificity metrics frequently exceeding 85% in late-stage clinical settings. Ultimately, confirmatory methods such as TRUS-guided biopsy, MRI-targeted biopsy, and histopathological assessment provide the highest diagnostic accuracy, with histopathology nearing the ideal sensitivity and specificity.

Taking together, these results demonstrate a definite trend toward more integrated approaches to diagnosis while using less invasive screening techniques in synergy with advanced imaging and confirmatory tissue analyses for purposes of improving diagnostic accuracy and avoiding unneeded invasive procedures.

The confidence interval findings depict a clear trade-off between accessibility and diagnostic accuracy, highlighting a significant variation in the consistency and reliability of PCa diagnostic techniques. Traditional screening techniques (PSA, DRE) displayed wider ranges and weaker specificity indicating a higher risk of false positives. On the other hand, more advanced biomarkers (PHI, PCA3, 4KScore, SelectMDx) indicate better sensitivity and specificity with narrower intervals, suggesting increased diagnostic stability and capacity for detection. Imaging modalities showed an even narrower confidence interval along with high sensitivity, highlighting their potential for even better diagnostic accuracy. Confirmatory techniques (MRI-targeted biopsy, histopathology) indicate the most reliable diagnostic performance.

Table 4 summarizes the comparative analysis of all the modalities for detecting prostate cancer discussed in this chapter. The preferred modality for research application, advantages, and limitations of each method are also highlighted. A crucial consideration is the balance between cost, invasiveness, and diagnostic accuracy. PSA and DRE tests have low costs with minimal invasiveness; hence these methods are preferred in large-scale academic and epidemiological studies due to their availability, low cost, and ease of administration.

These methods are chiefly constrained by low specificity and sensitivity, which results in overdiagnosis and unnecessary follow-up. Still, PSA derivatives (PHI and 4Kscore) as well as urine-based biomarkers have better specificity and clinically significant prostate cancer detection; hence they would be more suitable for risk stratification studies. Moderate costs plus limited availability may preclude their use in resource-poor settings.

Liquid biopsy and mpMRI are state-of-the-art methodologies with their respective benefits that give broad molecular information plus imaging data comprehensively. These modalities are very important for translational research multi-omics studies as well as precision diagnostics because of deep insights into tumor biology plus clinically relevant cancer identification; however high costs specialized infrastructure requirements will restrict their availability in resource-limited settings for large population-based studies.

TRUS-guided biopsy, MRI-targeted biopsy, and histopathology confirmatory modalities remain essential for definitive diagnosis. These are critical components in clinical validation studies despite their invasiveness associated with procedural risks. It is observed that less invasive cost-effective methods would be preferred for large-scale research applications while advanced invasive techniques would be more appropriate for precision-oriented clinical investigations thus underscoring the need for a combined context-specific diagnostic approach. The comparative cost analysis reveals significant differences in cost for these diagnostic techniques in South Africa, with the more affordable techniques costing approximately R1500 or less per test and the more advanced ones costing between R1500–R10,000 (moderate) and the sophisticate techniques potentially costing over R10,000.

## 4. Discussion

Table 2, Table 3 and Table 4 in this comparative review of prostate cancer detection methods exhibits an unending and clinically significant discord between diagnostic accuracy, invasiveness, cost, and research applicability. At a basic level, screening tools like prostate-specific antigen testing (PSA) and digital rectal examination still serve as primary alternatives because of their low costs, ease of administration, broad availability in low- and middle-income countries including several regions in Africa and South Africa [46,62,154].

The dominance of DRE and PSA in both the clinical field as well as research studies is more due to convenience than any claims of better performance. As clearly shown in Table 2, even though PSA testing has relatively high sensitivity, its considerable lack of specificity translates into relatively high false-positive rates with resultant overdiagnosis and unnecessary biopsy [24,26,35]. Similarly, although DRE is cheap, it does not have good sensitivity for picking up early-stage cancers and highly depends on the skill of the operator. From a research perspective however these limitations are somewhat offset by their usefulness in large-scale epidemiological and population-based studies where accessibility sample size is very critical [169,170].

On the other hand, the second-line and complementary biomarker approaches to prostate cancer such as PSA derivatives (PHI and 4Kscore) plus urine-based markers like PCA3 and TMPRSS2–ERG have made significant strides in risk stratification and diagnostic specificity. The data presented highlight that these methods work particularly well when distinguishing clinically significant malignancies from indolent forms of cancer, this being a major critique against PSA-based screening [138,144,156,171].

Their moderate cost and limited availability, especially in resource-poor settings hinder widespread adoption. It brings about a huge gap: even though these instruments are scientifically superior on many fronts they are not yet universally applicable [102,158,172,173]. From an academic point of view, these biomarkers PCA3 and TMPRSS2–ERG fit well into case–control studies for validation research within precision medicine but become impractical to use in large-scale population screening under resource constraints. Also, the differences in sensitivity seen between studies point to the need for better standardization and testing in different groups, especially African populations that are not well-represented in global studies [149,174,175]. Imaging methods add a lot of detail to the diagnosis process.

As shown by the information in Table 2, Table 3 and Table 4, transrectal ultrasound (TRUS) is often used due to its easy access and guiding role in biopsy but has relatively low sensitivity for clinically significant tumors [65,150,152]. On the other hand, multiparametric MRI (mpMRI) shows much better sensitivity and specificity, thus helping with better visualization of tumor location and aggressiveness. The current literature supports mpMRI as a new diagnostic tool that can reduce unnecessary biopsies while increasing the detection of clinically important conditions [150,153,176]. The mpMRI method called expensive highlights a big problem: it needs infrastructural resources to function properly. In many African nations, access to mpMRI is frequently restricted to tertiary or private healthcare facilities which limits its possible effectiveness in wider public health programs [177,178]. From a research perspective, mpMRI is very useful for studies that focus on accuracy in diagnosis as well as imaging-related investigations but its cost and technical requirements make it difficult to use widely among large cohort studies [179,180].

Also, new imaging methods like PSMA PET/CT increase diagnostic accuracy more at stages of disease and recurrence yet their high costs and unavailability restrict them mostly to advanced research projects plus special clinical settings [176,181,182]. Liquid biopsy is a very hopeful and increasingly growing testing method shown in discussions under tables one two and three. Its main benefit comes from being able to get easy access repeatedly to molecular and genomic information about tumors, making it very attractive for multi-omics as well as translational studies [183,184,185]. Compared with normal tissue biopsy liquid biopsy for prostate cancer reduces stress on patients while allowing active tracking of disease progress.

Its critiques arising from such comparisons indicate that, despite its substantial theoretical and research potential, liquid biopsy faces challenges due to its high costs, lack of sufficient standardization, and its incomplete integration into standard clinical practices [139,144,186,187,188]. The sensitivity of liquid biopsy may vary with tumor burden and methodological differences, indicating the need for further validation. Thus, though liquid biopsy is a good candidate for advanced academic research, particularly in genomics and personalized medicine, its current practical use in broad clinical or population-based settings is limited [189,190,191].

As per the data provided (Table 2, Table 3 and Table 4), even after advances in non-invasive and minimally invasive techniques, biopsy along with histopathological assessment still stands out as the definitive gold standard for diagnostic purposes. Although TRUS-guided biopsy is commonly used in clinical practice, it has some drawbacks such as the chance of sampling error and risk of infection [146]. On the other hand, MRI-targeted biopsy has been found to improve detection rates for clinically significant cancers [192]. Results analyzed via histopathology, especially with Gleason scoring, provide key information about how aggressive a tumor is, which helps with treatment decision-making [193]. However, since these procedures for prostate cancer are invasive (TRUS-guided biopsy and MRI-targeted biopsy) they cannot be considered suitable for screening applications. From a research viewpoint such methods are crucial for clinical validation outcome assessment as well as setting up diagnostic standards. With that being noted, the invasive nature of these techniques plus their risks limit their use in exploratory or large-scale screening studies [84,137,194,195].

A major theme that comes out of what the tables show is the basic trade-off between cost and how accurately something can be diagnosed. Cheap tests like prostate-specific antigen (PSA) testing and digital rectal examination (DRE) are common but not very precise. More accurate ways of diagnosis, such as using multiparametric magnetic resonance imaging (mpMRI), Positron Emission Tomography with Prostate-Specific Membrane Antigen (PSMA PET), or liquid biopsy, are usually more expensive and less available. This contrast is particularly evident in low- and middle-income countries where healthcare systems grapple with financial constraints and the need for high-quality diagnostics. From the literature, a tiered or integrated diagnostic approach is the most practical: starting with screening using cheap methods, then applying more specific biomarkers or imaging, and finally confirming through biopsy. This layered diagnostic strategy improves cost-effectiveness as well as diagnostic accuracy at the same time.

Despite the substantial mortality burden imposed by prostate cancer on African populations and their disproportionate incidence, knowledge regarding driver factors that might elucidate these contrasts is still limited. Resolving this gap is therefore imperative not only for Africa but also to inform the global understanding of prostate cancer epidemiology and its determinants among the fastest-growing population worldwide. A methodological gap exists in identifying actionable research domains pertaining to the specific context of African populations.

A major limitation across this reviewed literature is the substantial lack of diagnostic data pertaining to the South African population; a key indicator that this population continues to be underrepresented in biomarker validation studies and genomic research even though there is evidence that men of African ancestry have a higher incidence of PCa. Most molecular biomarker validations, sensitivity estimates, specificity values, and diagnostic thresholds have been generated primarily from populations in Europe, North America, or Asia, which restricts their direct relevance to African contexts. The effectiveness of new methods like liquid biopsy, mpMRI, PSMA PET/CT, PHI, and 4Kscore in African healthcare systems and genetically varied African populations has also not been thoroughly studied. These limitations thus make it harder to compare studies and may impact reproducibility and clinical use; especially in settings with limited resources. Therefore, some of these methods require more validation, protocol harmonization and extensive research before they can reach universal standardization despite their promising diagnostic capabilities.

## 5. Conclusions

The comparative analysis reveals that no individual diagnostic approach is adequate when used alone. Rather, each method fulfills a distinct function within a comprehensive diagnostic MODEL informed by clinical objectives, available resources, and research aims. Techniques that are less invasive and more cost-effective are particularly suited for broad population and epidemiological investigations, whereas more sophisticated imaging and molecular methodologies are better aligned with the principles of precision medicine and mechanistic research. A persistent challenge, especially in resource-limited environments, is to reconcile the divide between innovation and accessibility, thereby ensuring that advancements in prostate cancer diagnostics lead to fair enhancements in both research findings and clinical results.

Future approaches ought to focus on the creation and application of standardized, minimally invasive and reasonably priced diagnostic techniques that are practical in low-resource settings. To improve detection while diminishing reliance on expensive infrastructure and technology, a greater focus must be placed on verifying accessible biomarker-based assays and streamline imaging techniques. Additionally, improving healthcare worker training, expanding access to screening programs, strengthening healthcare infrastructure, and incorporating tiered diagnostic pathways that combine targeted advanced testing with affordable screening may improve early diagnosis and lower prostate cancer mortality.

## Figures and Tables

**Table 1:** Critical appraisal tools used in this study for quality assessment and their applicability to different designs.

Appraisal Tool	No. of Studies	Type of Study Assessed	Criteria Evaluated
Quality Assessment of Diagnostic Accuracy Studies (QUADAS-2)	56 [28,29,34,35,36,37,38,39,40,41,42,43,44,45,46,47,48,49,50,51,52,53,54,55,56,57,58,59,60,61,62,63,64,65,66,67,68,69,70,71,72,73,74,75,76,77,78,79,80,81,82,83,84,85,86,87,88,89]	Diagnostic accuracy, biomarker validation, and imaging perfomance	Patient selection, index test, reference standard, flow and timing, applicability concerns
Newcastle–Ottawa Scale (NOS)	8 [90,91,92,93,94,95,96,97]	Cohort, case–control, epidemiological, comparative clinical	Selection of participants, comparability of groups, exposure/outcome assessment
Joanna Briggs Institute (JBI)	54 [98,99,100,101,102,103,104,105,106,107,108,109,110,111,112,113,114,115,116,117,118,119,120,121,122,123,124,125,126,127,128,129,130,131,132,133,134,135,136,137,138,139,140,141,142,143,144,145,146,147,148]	Narrative, systemic, and scoping	Methodological quality

**Table 2:** A Summary of prostate cancer diagnostic techniques, clinical use and insights.

Method	Clinical Use	Notes	References
**Digital Rectal Exam (DRE)**			
DRE	Screening	Often combined with PSA for initial screening.	[45,46,47,48]
**Prostate-Specific Antigen (PSA) and Derivatives**			
PSA	Screening	Most common first-line screening test. Sensitivity and specificity is highly dependent on the standard cutoff (≥4.0 ng/mL).	[35,98,99]
Free/total PSA ratio	Screening/risk assessment	Used to improve PSA diagnostic accuracy.	[49,50,93]
PSA velocity (PSAV)/density (PSAD)	Detection/monitoring	Complementary metrics that help quantify the risk of clinically significant prostate cancer by integrating serum PSA concentration with prostate volume at a standard cutoff of 0.15 ng/mL for PSAD and 0.75 ng/mL for PSAV.	[51,52,53]
Prostate Health Index (PHI)	Screening/risk stratification	Utilized as a reflexive examination for males within the prostate-specific antigen (PSA) “gray zone” range of 2–10 ng/mL, aimed at minimizing the likelihood of superfluous biopsy procedures.	[36,37,38,39,90]
4Kscore test	Screening/risk prediction	The 4Kscore test is a blood assay authorized by the FDA, designed to assess the likelihood of detecting aggressive, high-grade prostate cancer, characterized by a Gleason score exceeding 7, in biopsy specimens.	[54,55,101,105,106]
**Prostate Cancer Gene and Urine Tests**			
PCA3 test	Screening/risk assessment	The efficacy of the PCA3 test is contingent upon the specific “score” (cutoff) utilized to suggest a biopsy.	[35,40,41,91,100,101]
TMPRSS2-ERG gene fusion test	Molecular detection	Represents a highly specific molecular assay designed to identify a genetic rearrangement predominantly associated with prostate cancer cells.	[42,43,44,92,102,103,104]
SelectMDx/ExoDx urine tests	Detection/risk stratification/biopsy guidance	Provide a means of “biopsy avoidance” specifically aimed at identifying aggressive prostate cancer. These assays are instrumental in aiding clinical decision-making for men who present with elevated prostate-specific antigen (PSA) levels.	[56,57,58,59,60,107,108,109,110]
**Liquid Biopsy and Circulating Biomarkers**			
Circulating Tumor DNA (ctDNA)	Detection/monitoring/guidance	Blood-derived circulating tumor DNA (ctDNA) exhibits a high degree of specificity for the monitoring of advanced prostate cancer. However, it is characterized by a significantly low sensitivity in early-stage disease. In contrast, urine-derived circulating tumor DNA (utDNA) reveals enhanced sensitivity for the early detection of prostate cancer.	[61,111,112,113,114]
Circulating Tumor Cells (CTCs)	Prognosis/monitoring	Traditionally, investigations into Circulating Tumor Cells (CTCs) have concentrated on their presence in blood; however, the identification of CTCs in urine is gaining traction as a novel avenue for the non-invasive detection and monitoring of prostate cancer (PCa).	[61,94,115,116,117]
Exosomes	Detection/biopsy guidance	Exosomes, which are vesicles measuring between 30 and 120 nanometers, play a significant role in the progression of prostate cancer (PCa). They function as “double-edged swords” due to their dual capacity to promote metastasis and confer drug resistance.	[62,63,64,118,119,120,121,123,124,125]
**Imaging Techniques**			
Transrectal Ultrasound (TRUS)	Detection/biopsy guidance	Serves predominantly as an instrument for guiding biopsies rather than functioning as an independent diagnostic modality.	[65,66,67,68,69,126,127,128]
Multiparametric MRI (mpMRI)	Detection/biopsy guidance	The current standard for the non-invasive detection and local staging of prostate cancer heavily depends on the precision afforded by the PI-RADS (Prostate Imaging-Reporting and Data System) score. High PI-RADS scores = more aggressive forms of the disease.	[70,71,72,73,74,95,129]
PSMA PET/CT Imaging		Classified as a non-invasive imaging modality, distinct from liquid biopsy, as it does not necessitate biological specimens such as blood or urine for its evaluation.	[75,76,77,78,79,80,130,131,132]
**Tissue Sampling and Pathology**			
TRUS-guided biopsy	Confirmation/biopsy guidance	Serves primarily as a technique for confirmation and biopsy guidance, rather than functioning as an independent screening tool. The diagnostic accuracy of this method is significantly dependent on whether it is conducted as a systematic (randomized) biopsy or as a targeted biopsy, often integrated with MRI findings.	[81,82,133,134,151,152]
MRI-targeted biopsy	Confirmation	It is frequently utilized in conjunction with systematic sampling to yield the most dependable validation of prostate cancer.	[128,129,130]
Histopathology (Gleason Scoring)	Confirmation/grading	Represent the benchmark for the validation of prostate cancer diagnoses.	[83,84,96,137]

**Table 3:** Comparative overview of diagnostic techniques and their clinical performance.

Method	Test/Sample Type	Clinical Use	Sensitivity (%)	Specificity (%)	Confidence Interval (95%)	References
**Digital Rectal Exam (DRE)**						
DRE	Physical examination	Screening	28.6–53.2	59–91	55–84	[45,46,47,48]
**Prostate-Specific Antigen (PSA) and Derivatives**						
PSA	Blood	Screening	21–44	91–94	18–45	[35,98,99]
Free/total PSA ratio	Blood	Screening/risk assessment	33–41	85–96	45–74	[49,50,93]
PSA velocity/density	Blood + prostate volume	Detection/monitoring	PSAD: 60–74 PSAV: 59	PSAD: 61–70 PSAV: 60	50–76	[51,52,53]
Prostate Health Index (PHI)	Blood	Screening/risk stratification	~95	16–36	55–79	[36,37,38,39,90]
4Kscore test	Blood	Screening/risk prediction	94–97	~40	56–83	[54,55,101,105,106]
**Prostate Cancer Gene and Urine Tests**						
PCA3 Test	Urine	Screening/risk assessment	47–69	65–76	66–84	[35,40,41,91,100,101]
TMPRSS2-ERG Gene fusion test	Urine	Molecular detection	23.5–49	86–100	80–97	[42,43,44,92,102,103,104]
SelectMDx/ExoDx urine tests	Urine	Detection/risk stratification/biopsy guidance	MDx: ~81–91 ExoDx: ~92	MDx: ~52–70 ExoDx: ~34–54	56–84	[56,57,58,59,60,107,108,109,110]
**Liquid Biopsy and Circulating Biomarkers**						
Circulating tumor DNA (ctDNA)	Blood/emerging for urine	Monitoring/therapy guidance detection/biopsy guidance	Blood: 20 (Early)–68 (Adv) Urine: 33–50	Blood: 95–100 Urine: 88–94	75–97	[61,111,112,113,114,138,139,140,141,142]
Circulating tumor cells (CTCs)	Blood/emerging for urine	Prognosis/monitoring	Blood Localized: 8–59 Metastatic: 44–79 Urine: 30–50	Blood Localized: 88–96 Metastatic: 90–98 Urine: 85–93	70–93	[61,94,115,116,117,143,144]
Exosomes	Blood/urine	Detection/biopsy guidance	Urine: 83–86 BLOOD: ~84	Urine: 88 BLOOD: ~86	65–93	[59,85,86,120,145]
**Imaging Techniques**						
Transrectal ultrasound (TRUS)	Imaging	Biopsy guidance Risk stratification Detection Biopsy improvement	Greyscale: ~20–50 AI-Enhanced: ~86–92.5 MicroUS: ~87–94 CETRUS: ~54–79.3	Greyscale: ~61–89 AI-Enhanced: ~68.1–92.1 MicroUS: ~22–31 CETRUS: ~42–95	55–74	[65,66,67,68,69,126,127,128]
Multiparametric MRI (mpMRI)	Imaging	Detection/biopsy guidance	~87–91	~30–64	66–93	[70,71,72,73,74,95,129]
PSMA PET/CT imaging	Imaging	Detection/monitoring/confirmation	Primary: 82–97 Localized: 71–82 Nodal: 42–57 BCR: 60–97 Metastasis: ~85	Primary: 49–67 Localized: 67–92 Nodal: 94–98 BCR: 86–99 Metastasis: ~98	76–97	[75,76,77,78,79,80,130,131,132]
**Tissue Sampling and Pathology**						
TRUS-guided biopsy	Tissue	Confirmation/biopsy guidance	12 core: 48–60 Fusion: 83–94	12 core: 70–96 Fusion: 66–80	95–100	[81,82,87,88,133,146]
MRI-targeted biopsy	Tissue	Confirmation	~80–94	~66–94	96–100	[87,89,136,153]
Histopathology (Gleason scoring)	Tissue	Confirmation/grading	~92–97	~91–100	98–100	[97,147,148]

**Table 4:** Evaluation of diagnostic methods based on practical research considerations.

Method	Invasiveness	Cost	Advantages	Limitations	Suitability for Research	References
DRE and PSA	Minimal	Affordable	Low cost, highly accessible.	Low specificity leads to excessive number of diagnoses and unnecessary biopsies.	Crucial for longitudinal and population-focused epidemiological studies.	[35,154,155]
PHI	Minimal	Moderate	Demonstrates 3× greater specificity than PSA testing alone	Requires specialized immunoassay systems.	An area of active research for validating findings across various ethnic groups.	[38,39]
4Kscore test	Minimal	Expensive	Elevated NPV surpassing 95% suggests great capacity to rule out the existence of aggressive malignancies.	Expensive and has complex algorithms.	Important for health economics and research on risk prediction.	[101,106,156]
Urinary biomarkers (PCA3, TMPRSS2-ERG)	Non-invasive	Moderate	High specificity.	May require prostate massage and has variable specificity.	Useful for biomarker discovery and molecular studies.	[157,158,159,160,161,162]
SelectMDx ExoDx	Non-invasive	Moderate Expensive	Help avoid ~50% biopsies. No DRE required, at home collection available.	DRE before collection to be most effective. Potential for out-of-pocket costs.	Focus on pre-MRI triage and clinical use. Emerging; key for exosome biology and non-invasive molecular tracking.	[56,58,59,107]
Liquid biopsy (ctDNA, CTCs, exosomes)	Minimal	Expensive	Provides genomic and molecular data plus real time monitoring.	Expensive and has limited standardization.	Great for multi-omics and translational studies; critical for precision oncology and treatment resistance studies.	[139,144,163,164]
Transrectal ultrasound (TRUS)	Minimal	Moderate	Widely available and guides biopsy	Has very low sensitivity for early onset cancer, may miss tumors.	Great for imaging-based studies.	[24,127,146,165]
Multiparametric MRI (mpMRI)	Non-invasive		Reduces unnecessary biopsies.	Requires certain skills and expertise. Limited standardization.	Valuable for diagnostic accuracy research.	[150,154,166,167]
PSMA PET/CT imaging	Non-invasive	Expensive	Highest level of accuracy for staging and assessing recurrence, capable of identifying micrometastases.	Limited availability.	Great for advanced and precision medicine studies.	[75,76,77,78,79,80,130,131,132]
TRUS-guided biopsy	Invasive	Moderate to expensive	Provides tissue confirmation.	Runs a risk of infection due to invasive nature.	Valuable for validating and clinical research.	[75,76,77,78,79,80,130,131,132]
MRI-targeted biopsy	Invasive	Expensive	Great for detection of essential cancer.	Needs MRI equipment.	Useful for advanced diagnostic research.	[135,136,168]
Histopathology (Gleason scoring)	Invasive	Moderate	Diagnosis is definite and can grade tumor aggressiveness	Requires invasive biopsy.	Great for confirming clinical studies.	[83,84,96,137]

## Data Availability

All datasets associated with this publication are presented in this manuscript.
